# Energy Disaggregation Using Elastic Matching Algorithms

**DOI:** 10.3390/e22010071

**Published:** 2020-01-06

**Authors:** Pascal A. Schirmer, Iosif Mporas, Michael Paraskevas

**Affiliations:** 1 Communications and Intelligent Systems Group, School of Engineering and Computer Science, University of Hertfordshire, Hatfield AL10 9AB, UK; i.mporas@herts.ac.uk; 2 Computer Technology Institute and Press “Diophantus”, Dept of Electrical and Computer Engineering, University of Peloponnese, 221 00 Tripoli, Greece; mparask@cti.gr

**Keywords:** non-intrusive load monitoring (NILM), energy disaggregation, elastic matching algorithms, dynamic time warping, minimum variance matching

## Abstract

In this article an energy disaggregation architecture using elastic matching algorithms is presented. The architecture uses a database of reference energy consumption signatures and compares them with incoming energy consumption frames using template matching. In contrast to machine learning-based approaches which require significant amount of data to train a model, elastic matching-based approaches do not have a model training process but perform recognition using template matching. Five different elastic matching algorithms were evaluated across different datasets and the experimental results showed that the minimum variance matching algorithm outperforms all other evaluated matching algorithms. The best performing minimum variance matching algorithm improved the energy disaggregation accuracy by 2.7% when compared to the baseline dynamic time warping algorithm.

## 1. Introduction

In recent years, the world energy demand has increased due to the population growth and economic development [1] and it is expected that it will further increase in the next decades [2]. The energy demand worldwide is annually increasing both in the residential and the industrial sector with households consuming approximately 40% of the world’s consumed energy [3,4]. The technological development of the last decades has led to low costs for buying electrical appliances and the automation of tasks and procedures both in industry and in households, thus it is estimated that the electric power needs will further grow and the average number of electrical appliances per household will significantly increase within the next two decades [4]. It is estimated that approximately 20% of the energy consumed in the residential sector could be saved by changing consumers’ behavior and by improving the existing poor operational strategies [5,6]. Moreover, the development of smart grids and energy demand management systems as well as the fluctuation of power generation due to the increasing percentage of power generated by renewable energies units can confine the issue of annually increasing energy demands [7,8]. These changes in energy demand and generation are challenging for network operators and power generation units, since power needs are becoming less stable and less predictable while at the same time energy demand increases [9,10]. To address the above mentioned challenges, precise monitoring of electrical energy consumption in the residential sector is needed [10], as well as proper energy demand prediction and management [9]. At the moment energy consumption monitoring is mostly done by measuring the aggregated energy consumption in the form of monthly bills and therefore does not address the above-mentioned issues.

The measurement of energy consumption is performed using smart meters (SM). Smart meters measure the voltage drop over a device or a circuit and the current flowing through it at a predefined sampling rate with the sampling period varying from milliseconds to minutes [11]. The lower the sampling period, the more accurate temporal information of the energy consumption signal is recorded, however high sampling frequency increases the amount of data acquired per time unit and also requires hardware supporting high sampling frequency A/D conversion, which in general increases the cost of hardware [12] and might not lead to better disaggregation results [13]. Most commercial smart meters must use a sampling rate in the order of seconds for the transmission and storage of energy data for several months or years to be feasible and to keep the corresponding hardware costs relatively low.

Energy consumption should not be monitored at a household level but rather at the device level, in order to detect faulty device operation and inefficient or suboptimal operational strategies and thus maximize improvements in terms of energy savings as shown in [14]. To measure energy consumption at device level, energy usage has to be measured either for each device separately using one smart meter per device or the household aggregated energy consumption (sum of energy consumption from several devices measured at one central point e.g., the power inlet of a household) has to be disaggregated to device level using computational algorithms. When using only one sensor (smart meter) to disaggregate the total consumed energy and to extract energy consumption on the appliance level the task is called non-intrusive load monitoring (NILM) as introduced in [15]. In the NILM approach the energy disaggregation task is expressed as a single-channel source separation problem, where the smart meter is the only input channel measuring the total power consumption and the goal is to find the inverse of the aggregation function to calculate the energy consumption per device. In intrusive load monitoring (ILM) one smart meter per device is used, thus measuring the energy consumption directly from each device. Compared to ILM, NILM has the advantage of requiring less hardware (ILM uses one smart meter per device which is impractical for most households) as well as meets consumers’ acceptability with respect to privacy conserving [7,16]. NILM approaches assume that there is a single observation (smart meter measurements) and multiple unknowns (power consumption of electrical devices) making the disaggregation problem highly under-determined and difficult to solve without any further constraints.

Several approaches for NILM have been proposed in the literature. In these approaches one or multi-state electrical devices have been modeled by finite-state machines, i.e., with steady energy consumption behavior per operational state [15,17,18]. In contrast to one/multi-state devices, there is no established approach in detecting appliances with continuous power consumption or with non-linear behavior and a highly-varying power signature [19,20]. Researchers have addressed this issue by using high frequency features or wavelets to detect transient device behavior, however, these have the drawback of a higher cost in hardware and an increased computational power needed [12,20,21]. Therefore most approaches use disaggregation algorithms with sampling rates in the order of seconds to minutes, in addition with temporal information (e.g., factorial hidden Markov models (FHMM) [22,23]) to identify appliances with varying power consumption [12,24]. Furthermore, special filtering techniques (e.g., Kalman filters [25]) with time-varying coefficients and probabilistic approaches using appliance grouping [26] have been proposed to address the issue of modeling devices with continuous or non-linear characteristics. The NILM approaches can briefly be classified into methods with and without source separation (SS). Approaches without SS are based on the decomposition of the aggregated signal to a sequence of feature vectors, which will be classified to device labels by a machine learning (ML) algorithm (e.g., artificial neural networks (ANN) [27], cecision trees (DT) [28], hidden Markov models (HMM) [22], k-nearest neighbors (KNN) [29], support vector machines (SVM) [30]) or by a predefined set of rules and thresholds [31,32]. Furthermore, recent research in deep learning and big data has led to a significant increase of use of data-driven approaches using large scale datasets (e.g., AMPds [33]). Approaches based on convolutional neural networks (CNNs) [34,35,36], recurrent neural networks (RNNs) [37,38] and long short time memories (LSTM) [37,39] have been proposed in the literature, while denoising autoencoders (dAEs) [40] and gate recurrent units (GRUs) [36] have also been used. Approaches with SS are based on single-channel source separation algorithms (e.g., non-negative matrix factorization [41], sparse component analysis [42]) to extract the consumption of each device from the aggregated signal by using additional constraints (e.g., sparseness or sum-to-one [43]) during the optimization procedure. The features extracted from the aggregated signal in approaches with and without SS strongly depend on the sampling frequency, with either macroscopic (for low sampling frequency) or microscopic (for high sampling frequency) features being extracted. Macroscopic features are mainly active and reactive power, while statistical values from the active or reactive power (e.g., mean, median, variance or energy) can be estimated as well [44]. Microscopic features can be current harmonics or transient energy [31,45] and require high-sampling frequency to be calculated (1 kHz and above).

In addition to the above-mentioned machine learning-based NILM solutions, approaches using template matching have been proposed. More specifically, in [46] dynamic time warping (DTW) was used to detect transient signatures for NILM and a weighted DTW was proposed and evaluated for different sampling frequencies. In [47] a hybrid detection approach utilizing FHMMs and DTW-based iterative subsequence clustering was introduced for generating subsequences to refine initial estimates provided by the FHMM. In [48] load disaggregation was performed using subsequence searching by utilizing DTW and iteratively disaggregate one appliance at a time in order of decreasing energy consumption. In [49] a DTW-based pattern matching approach was proposed and its performance was compared to HMMs and DTs.

In this paper, an architecture based on elastic matching algorithms for non-intrusive load monitoring is proposed. In contrast to machine learning-based approaches which require significant amount of data to train a model, elastic matching-based approaches do not have any model training process but perform recognition using template matching. Except for a few papers [46,47,48,49] that have used only the DTW algorithm for NILM, no previous work on the evaluation of elastic matching algorithms for energy disaggregation has been published in the literature. In the proposed architecture, excluding DTW, several other elastic matching algorithms such as the global alignment kernel, the soft dynamic time warping, the minimum variance matching and the all common subsequences have been used. The remainder of this article is organized as follows. In Section 2 five different elastic matching algorithms are reviewed. In Section 3 the proposed architecture for energy disaggregation using elastic matching is presented. In Section 4 and Section 5 the experimental setup and evaluation results are described, respectively. In Section 6 we conclude this work.

## 2. Elastic Matching Algorithms

In the context of energy disaggregation five different elastic matching algorithms, which can be used to compare any two time series of unequal lengths, are reviewed. These are the DTW algorithm, which has been used before in the NILM task [46,47,48,49], as well as the global alignment kernel (GAK), the soft dynamic time warping (sDTW), the minimum variance matching (MVM) and the all common subsequences (ACS), which have not been used before in the NILM task. GAK, sDTW, MVM and ACS algorithms were chosen as they offer additional degrees of freedom on the warping path [50,51,52] comparing to the DTW algorithm.

Considering the aggregated power consumption signal $P_{agg}\left(t\right)\forall t:t\in \left\{1,\cdots ,T\right\}$ acquired by a smart meter let $P_{a}=\left[p\left(i\right)p\left(i+1\right)\cdots p\left(i+N\right)\right]$ be a sequence of length *N* where p(i) is the $i^{th}$ sample of $P_{agg}$ and let $P_{b}=\left[p\left(j\right)p\left(j+1\right)\cdots p\left(j+M\right)\right]$ be a second sequence of length *M* where p(j) is the $j^{th}$ sample of $P_{agg}$ and N<M. Furthermore let $\Delta \left(P_{a},P_{b}\right)={\left[\delta \left(p_{a}^{n},p_{b}^{m}\right)\right]}_{i,j}\in R^{NxM}$ be an arbitrary cost matrix, where δ(·) is a distance metric e.g., Euclidean distance, Manhattan distance or Kullback–Leibler (KL) distance and $\langle A,\Delta \left(P_{a},P_{b}\right)\rangle$ being the inner product of matrix *A* with the cost matrix $\Delta (P_{a},P_{b})$, where *A* is an alignment matrix with $A_{n,m}$ being the alignment score between the $n^{th}$ and the $m^{th}$ element of $P_{a}$ and $P_{b}$ respectively.

### 2.1. Dynamic Time Warping

Based on the above, using the cost matrix $\Delta (P_{a},P_{b})$ and the different alignment matrices *A*, $DTW(P_{a},P_{b})$ is the minimum accumulated cost between $P_{a}$ and $P_{b}$ for all possible warping paths in the (N,M) search space. Accordingly the minimum cost is defined as in Equation (1) and the recursive update rule for finding the optimal warping path is given in Equation (2) [51,53].

(1) $$DTW\left(P_{a},P_{b}\right):=\min_{A\in A_{n,m}}\langle A,\Delta \left(P_{a},P_{b}\right)\rangle ,$$

(2) $$D\left(n,m\right)=\delta \left(p_{a}^{n},p_{b}^{m}\right)+min\left\{\begin{array}{c} D(n-1,m)\\ D(n-1,m-1)\\ D(n,m-1) \end{array}\right.,$$

where $D\left(n,m\right)=\sum_{k=1}^{L}\delta \left(p_{a}^{n},p_{b}^{m}\right)$ is the accumulated cost associated with any warping path $a=(a_{1},a_{2},\ldots ,a_{k},\ldots ,a_{l})$ from (i,j) to (i+N,j+M) with path-length *L* and point $a_{k}=\left(n_{k},m_{k}\right)\in \left\{i,i+1,\cdots ,i+N\right\}\left\{j,j+1,\cdots ,j+M\right\}$. Furthermore the initial conditions for the accumulated cost are set as follows: D(0,0)=0, D(n,0)=∞ for n>0 and D(0,m)=∞ for m>0.

### 2.2. Global Alignment Kernel

Extending the previous definition of DTW in Section 2.1 the global alignment (GA) kernel is defined as the exponentiated soft-minimum of all alignments distances and can be written as in Equation (3) [50]

(3) $$k_{GA}^{\gamma }:=\sum_{A\in A_{n,m}}e^{-\langle A,\Delta \left(P_{a},P_{b}\right)\rangle /\gamma },$$

where γ>0 is the smoothing parameter of the kernel. Compared to DTW, $k_{GA}^{\gamma }$ incorporates the whole spectrum of costs $\langle A,\Delta \left(P_{a},P_{b}\right)\rangle$ and thus provides a richer representation than the absolute minimum of set *A*, as considered by DTW [50].

### 2.3. Soft Dynamic Time Warping

As described in [51] Equations (1) and (3) can be computed using a single algorithm. The generalized $min^{\gamma }$ operator, with the smoothing parameter γ≥0 can be written as in Equation (4) and is referred to as soft dynamic time warping $dtw_{\gamma }$.

(4) $$dtw_{\gamma }:=min^{\gamma }\left\{\langle A,\Delta \left(P_{a},P_{b}\right)\rangle \;A\in A_{n,m}\right\},$$

(5) $$min^{\gamma }\left\{a_{1},\cdots ,a_{n}\right\}:=\left\{\begin{array}{cc} min_{i\leq n}a_{i} & \gamma =0\\ -\gamma log\sum_{i=1}^{n}e^{-a_{i}/\gamma } & \gamma >0 \end{array}\right.,$$

where the original DTW score is recovered by setting γ=0, while for γ>0 a scaled version of GAK can be written as $dtw_{\gamma }=-\gamma \log k_{GA}^{\gamma }$.

### 2.4. Minimum Variance Matching

In contrast to DTW, sDTW and GAK, MVM tries not to find the optimal alignment between the two sequences $P_{a}$ and $P_{b}$, but also considers the alignment of subsequences. Thus MVM tries to find a subsequence $P_{a}^{{}^{\prime }}$ of length *N* such that $P_{b}$ best matches $P_{a}^{{}^{\prime }}$. To formally describe MVM the difference matrix *r* between the two sequences $P_{a}$ and $P_{b}$ and is defined as follows [52]:

(6) $$r=\left(r_{nm}\right)=\left(p_{a}^{n}-p_{b}^{m}\right).$$

Furthermore, $r_{nm}$ is treated as a directed graph with the following links [52]:

(7) $$r_{nm}↔r_{kl}\;\;\;where\;k-n=1\;and\;m+1\leq m+N-M.$$

Using Equations (6) and (7) the least-value path in terms of the linkcost and pathcost can be written as described by Equations (8) and (9).

(8) $$linkcost\left(r_{nm},r_{kl}\right)=\left\{\begin{array}{c} {\left(r_{kl}\right)}^{2}={\left(p_{b}^{k}-p_{a}^{n}\right)}^{2}\;\;\;if\;k=n+1\;and\;m+1\leq l\leq m+1\left(N-M\right)-\left(m-n\right)\\ \infty \;\;\;otherwise \end{array}\right.,$$

(9) $$linkcost\left(n,m\right)=\left\{\begin{array}{c} {\left(r_{nm}\right)}^{2}\;\;\;if\;k=n+1\\ \min(pathcost\left(n,m\right),pathcost\left(n-1,k\right)+linkcost\left(r_{(n-1)k},r_{nm}\right))\\ if\;2\leq i\leq M,n\leq k\leq n+N-M,k+1\leq j\leq k+1+(N-M)\\ \infty \;\;\;otherwise \end{array}\right..$$

### 2.5. All Common Subsequences

As proposed in [54] the number of all common subsequences $acs(P_{a},P_{b})$, of any two sequences $P_{a}$ and $P_{b}$, can be found using dynamic programming. Specifically let N(n,m) be the number of common subsequences then:

(10) $$N\left(n,m\right)=N\left(n-1,m-1\right)\cdot 2,\;\;if\;p_{a}^{n}=p_{b}^{m},$$

(11) $$N\left(n,m\right)=N\left(n-1,m\right)+N\left(n,m-1\right)-N\left(n-1,m-1\right),\;\;if\;p_{a}^{n}\neq p_{b}^{m},$$

and consequently $acs\left(P_{a},P_{b}\right)=N(|P_{a}\left|,\right|P_{b}\left|\right)$.

## 3. NILM Using Elastic Matching

Considering a set of *M*-1 known devices each consuming power $p_{m}$ with 1≤m≤M, the aggregated power $P_{agg}$ measured by the sensor will be

(12) $$P_{agg}=f\left(p_{1},\ldots ,p_{M-1},g\right)=\sum_{m=1}^{M-1}p_{m}+g=\sum_{m=1}^{M}p_{m},$$

where $g=p_{M}$ is a ‘ghost’ power consumption (noise) consumed by one or more unknown devices and *f* is the aggregation function. In NILM the goal is to find precise estimations ${\hat{p}}_{m},\hat{g}$ of the power consumption of each device *m* using an estimation method $f^{-1}$ with minimal estimation error and ${\hat{p}}_{M}=\hat{g}$, i.e.,

(13) $$\begin{array}{cc} \hat{P} & =\left\{{\hat{p}}_{1},{\hat{p}}_{2},\ldots ,{\hat{p}}_{M-1},\hat{g}\right\}=f^{-1}\left(P_{agg}\right)\\ \; & s.t.\;\;\underset{f^{-1}}{\mathrm{argmin}}\left\{{\left(P_{agg}-\sum_{m=1}^{M}{\hat{p}}_{m}\right)}^{2}\right\} \end{array}$$

**Figure 1:**
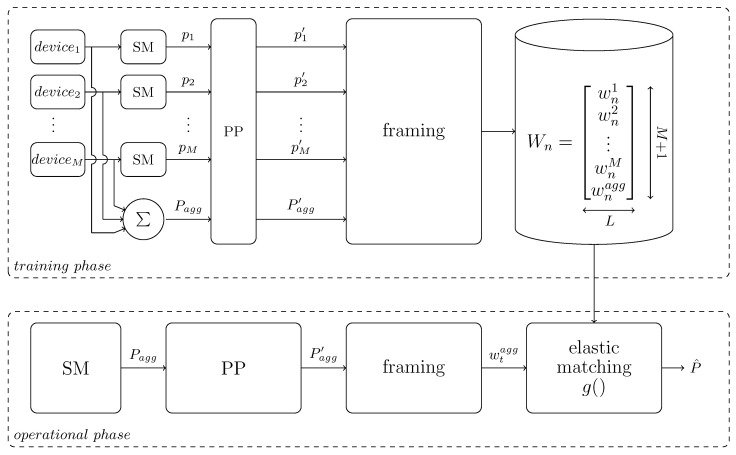
Block diagram of non-intrusive load monitoring (NILM) architecture using elastic matching. Smart meters are denoted with SM and preprocessing steps with PP.

In the proposed approach the minimization is performed using a database of power consumption signatures built from frames of the aggregated signal $P_{agg}$ and their corresponding ground-truth information for each appliance, providing estimates ${\hat{p}}_{m}$ for each $p_{m}$. The block diagram of the proposed NILM architecture is illustrated in Figure 1.

As illustrated in Figure 1 the proposed approach consists of three steps, namely preprocessing, framing and template matching using an elastic matching algorithm. During the training phase the energy consumption of each of the *M* devices, $p_{m}$, of a household and the aggregated consumption, $P_{agg}$, are recorded from smart meters (denoted as SM). The acquired measurements (*M*+1 time-synchronous signals) are preprocessed using a filter to remove outliers and static noise from the smart meters, frame blocked in frames $w_{n}^{m}$, $w_{n}^{m}\in R^{L}$, of constant length L=||w|| with 1≤n≤N being the number of frames and grouped, i.e., every stored aggregated energy consumption frame (reference frame) is stored together with the corresponding time-synchronous energy consumption frames of each of the *M* devices, into a table $W_{n}$, $W_{n}\in R^{(M+1)xL}$. Finally all tables $W_{n}$ are stored in a database $W:W_{n},\;1\leq n\leq N$. During the operational phase only the aggregated signal $P_{agg}$ is measured from a (central/main) smart meter. Similarly to the training phase, the aggregated signal $P_{agg}$ is initially preprocessed and frame blocked in frames of the same constant length L=||w||, with *t* being the number of the frame of the aggregated signal during operation. Each frame $w_{t}^{agg}$ is then compared against all aggregated power consumption reference frames $w_{n}^{agg}$ stored in the database *W* using an elastic matching algorithm g() and from the best matching reference frame the *M* device frames are used for numerical estimation, $\hat{P}={\hat{p}}_{m}$, of the power consumption of each of the *M* devices as described in Equations (14) and (15).

(14) $$k\left(t\right)=\underset{W:W_{n},1\leq n\leq N}{\mathrm{argmin}}\left\{g\left(w_{t}^{agg},w_{n}^{agg}\right)\right\},$$

(15) $${\hat{P}}_{t}=\left\{{\hat{p}}_{1}=\frac{1}{L}\sum_{L}w_{k(t)}^{1},{\hat{p}}_{2}=\frac{1}{L}\sum_{L}w_{k(t)}^{2},\cdots ,{\hat{p}}_{M}=\frac{1}{L}\sum_{L}w_{k(t)}^{M}\right\}.$$

In both the training and operational phase, only the active power samples of the device and aggregated signals were used since not all elastic matching algorithms can align multidimensional time-series data [52,54].

## 4. Experimental Setup

The NILM architecture using elastic matching presented in Section 3 was evaluated using the datasets, parameters and elastic matching algorithms described below.

### 4.1. Databases

To evaluate the proposed architecture the reference energy disaggregation dataset (REDD) [55] database has been used. The REDD database contains energy consumption recordings from home devices together with the aggregated energy consumption measurements from six households in the United States. Details of the datasets in the REDD database, one dataset per household, are tabulated in Table 1 with the number of appliances denoted in column ‘#App’ and the maximum number of appliances working in parallel denoted in column ‘#ParaApp’. The next three columns in Table 1 show the sampling period ‘$T_{s}$’, the duration ‘*T*’ in days, ignoring the gaps in the measurements [56], and the appliance types appearing in each evaluated dataset. The appliances type categorization is based on their operation as described in [17,57]. Previous publications [56,58,59] have excluded REDD-5 dataset from their experimental setup because of the significantly shorter duration of provided data compared to the rest of the REDD datasets, however in the present evaluation all six datasets have been used in order to evaluate the performance of the proposed architecture also under limited available training data conditions.

### 4.2. Preprocessing and Parametrization

During preprocessing the aggregated signal was initially processed by a median filter of five samples as proposed in [60] and then was frame blocked in frames of L=25 samples with overlap between successive frames equal to 15 samples. The optimal framelength was selected after grid search on a bootstrap subset from the REDD database, using the active power samples and DTW-based elastic matching as the baseline system. In detail the first five days from each REDD-x dataset were used, except for REDD-5 where only the first day was used, to create a bootstrap dataset and all results were calculated using estimation accuracy ($E_{ACC}$) as defined in [55]. The results are tabulated in Table 2.

As can be seen in Table 2 the highest average performance across all datasets was reached using a framelength of L=25 samples resulting in a disaggregation accuracy of 79.61%. In detail REDD-1,2,5 reached their highest performance using L=25 samples, while REDD-3,4,6 reached a slight higher accuracy for L=100/200 samples, but not significantly higher than L=25 samples, thus L=25 samples was selected as optimal frame length.

### 4.3. Elastic Matching Algorithms

For the elastic matching stage the five elastic matching algorithms presented in Section 2 were evaluated namely DTW, GAK, sDTW, MVM and ACS. The free parameters of each elastic matching algorithm were empirically optimized after grid search on a bootstrap training subset as described in Section 4.2. The best performance corresponding to the optimal values of each regression model is shown in bold. In detail all grid searches used as optimal framelength L=25 as estimated for DTW (baseline architecture) in Section 4.2. Firstly, two different restrictions on the DTW warping path were evaluated, namely the Sakoe and Itakura as proposed in [53,61]. The results are tabulated in Table 3.

As can be seen in Table 3 any restriction on the DTW warping path leads to a significant reduction of the energy consumption disaggregation accuracy with Itakura showing an average performance reduction of 5.8% and Sakoe of 6.8%, respectively. Based on the above evaluation results were calculated without any restrictions in the warping path. Secondly, different distance metrics, namely the Euclidean (Equation (16)), Manhattan (Equation (17)), Square (Equation (18)) and Kullback–Leibler (KL) (Equation (19)) were evaluated. These metrics for two *K*-dimensional signals $P_{a}$ and $P_{b}$ are given in Equations (16)–(19) and the evaluation results are tabulated in Table 4.

(16) $$\delta \left(P_{a},P_{b}\right)=\sqrt{\sum_{k=1}^{K}\left(p_{a}^{n}-p_{b}^{m}\right)\cdot \left(p_{a}^{n}-p_{b}^{m}\right)},$$

(17) $$\delta \left(P_{a},P_{b}\right)=\sum_{k=1}^{K}\left|p_{a}^{n}-p_{b}^{m}\right|,$$

(18) $$\delta \left(P_{a},P_{b}\right)=\sum_{k=1}^{K}{\left(p_{a}^{n}-p_{b}^{m}\right)}^{2},$$

(19) $$\delta \left(P_{a},P_{b}\right)=\sum_{k=1}^{K}\left(p_{a}^{n}-p_{b}^{m}\right)\cdot \left(\log p_{a}^{n}-\log p_{b}^{m}\right).$$

As can be seen in Table 4 there is no significant influence in terms of accuracy on the distance metric. However both Euclidean and Manhattan slightly outperform Square and KL, having the highest average performance for five out of the six bootstrap datasets, thus in the following evaluation all results are calculated using Euclidean distance. Regarding the free parameters of GAK, sDTW and MVM were selected using the bootstrap dataset of REDD-1 while using the optimal framelength L=25, with no restriction on the warping path and Euclidean distance metric as determined above. In detail the optimal values for the smoothing parameter γ of GAK and sDTW and the number of samples that can be left out by MVM were determined using grid search. The results are tabulated in Table 5.

As can be seen in Table 5 the optimal parameter values for the evaluated elastic matching algorithms are γ=10 for GAK, γ=5 for sDTW, while for MVM the number of samples left out were found to have no influence on the performance of MVM thus it was arbitrarily set to its default value v=10.

## 5. Experimental Results

The performance was evaluated in terms of estimation accuracy ($E_{ACC}$) considering device operation in state level with a double counting of errors as proposed in [55], i.e.,

(20) $$E_{ACC}=1-\frac{\sum_{t=1}^{T}\sum_{m=1}^{M}\left|{\hat{p}}_{t}^{m}-p_{t}^{m}\right|}{2\sum_{t=1}^{T}\sum_{m=1}^{M}\left|p_{t}^{m}\right|},$$

where *T* is the number of disaggregated frames and *M* the number of appliances including the ‘ghost’ device. The five different elastic matching algorithms described in Section 2 were evaluated on the REDD database using all houses and all available data. Specifically a 10-fold cross validation protocol was followed, with 90% of the data being used for building the signature database and 10% of the data for evaluating the proposed elastic matching-based NILM architecture. The evaluation results are tabulated in Table 6.

As can be seen in Table 6 MVM outperforms all other evaluated elastic matching algorithms across all datasets as well as on average increasing disaggregation accuracy approximately 2.7% resulting in an absolute average disaggregation accuracy of 80.93%. Furthermore sDTW offered a slight improvement with respect to the DTW baseline system with a performance increase of 0.8% and a total disaggregation accuracy of 78.95%. Moreover, GAK’s average performance was slightly lower than the baseline DTW (−1.0%), with the REDD-2 and REDD-5 datasets performing significantly lower than DTW. ACS was observed to perform significantly lower than DTW across all houses as well as in average, which is probably owed to the fact that ACS forces matching of subsequences and has neither a soft a margin as sDTW/GAK nor can it skip outliers like MVM [62]. It is worth mentioning that the energy disaggregation accuracy of the REDD-5 dataset is above 80% for both DTW and MVM despite the limited amount of available data for this household.

Furthermore results on the device level are presented for house two of the REDD database. REDD-2 was chosen as all appliances were metered over the whole recording period and there are no gaps in the measurements. For the purpose of direct comparison with previous studies we additionally tested our proposed methodology on five selected loads from the REDD database, so called deferrable loads, defined in [63]. These loads (reported as deferrable loads), namely the refrigerator, the lighting, the dishwasher, the microwave and the furnace (not available in REDD-2), were proposed as they contain a significant amount of the total consumed energy and were used in previous publications [56,63]. For evaluating estimation accuracy on device level Equation (20) is modified by eliminating the summation over *M* appliances resulting to Equation (21).

(21) $$E_{ACC}=1-\frac{\sum_{t=1}^{T}\left|{\hat{p}}_{t}^{m}-p_{t}^{m}\right|}{2\sum_{t=1}^{T}\left|p_{t}^{m}\right|}.$$

The results are tabulated in Table 7, with the last row presenting the average disaggregation accuracy computed according to Equation (21) and the second column presenting the percentage of the total energy consumed by each appliance.

As can be seen in Table 7 DTW in general offers good performance for appliances with one/multi-state behavior (e.g., refrigerator, microwave or dishwasher) and performs poorly for device operating for long duration and without many state changes (e.g., lighting or kitchen-outlets), which is in agreement with the evaluation results in [49]. MVM was found to improve the disaggregation accuracy of appliances with long operational duration due to its ability of matching subsequences without being restricted in aligning the corresponding first and last samples of the two sequences as in the case of DTW alignment. Furthermore as stated in [64] MVM allows the skipping of outliers that are present in the test series $w_{t}^{agg}$ and thus is able to handle noisy data better compared to DTW. In detail lighting and kitchen-outlets showed the largest improvements with 11.1% (10.5%) and 8.3%, respectively. Moreover the detection of ghost power, which usually appears in the aggregated signal and has a high variance due to possibly several unknown devices working in parallel was further improved achieving disaggregation accuracy of 90.96%.

The best performing MVM elastic matching algorithm is compared to other methods proposed in the literature that have been evaluated on the REDD database. It is worth mentioning that the number of datasets used across previous studies was not the same thus MVM performance has been calculated for each dataset setup (datasets 1,2,3,4,6; dataset 2; referable loads of dataset 2; fridge of dataset 2). Also the split of the data to training/test subsets is not the same in the literature thus only rough comparison is possible. The results are tabulated in Table 8.

As can be seen in Table 8 the best performing elastic matching algorithm MVM outperforms all other reported approaches on the REDD-1/2/3/4/6 dataset setup. Similarly the results of REDD-5 dataset setup showing the advantage of elastic matching over machine learning-based approaches when limited available training data exit. Considering the REDD-2 dataset setup with deferrable loads, which was initially proposed in [63], the proposed methodology using elastic matching outperforms all reported methodologies. The exception is the method of Makonin et al. [56] that utilized HMM sparsity, which performed 2.9% better than our proposed MVM, however the approach in [56] is specifically designed for deferrable loads and performances using all appliances of each house of the REDD database are not reported. Considering the latest deep learning techniques using CNNs, our MVM-based elastic matching approach performed 7.3% better for the fridge only REDD-2 dataset setup in [38].

For the purpose of direct comparison of the above-presented evaluation results with the previous evaluation of the DTW algorithm, the approach presented in [49] using a DTW and evaluated in houses REDD-1,2,6 was used. In detail, the approach presented in [49] uses a train/test data splitting, with the first week of every dataset used for training and the rest for testing as well as a lower sampling rate of 1 min, thus the results have been recalculated according to the setup of [49]. Furthermore the approach is event-based thus performance is measured using the $F_{1}$-score as defined in Equation (22),

(22) $$F_{1}=2\cdot \frac{TP}{2\cdot TP+FN+FP},$$

and a set of thresholds is used to decide if a device is operating within each frame or not. In Equation (22) TP, FN and FP are the True Positives, False Negatives and False Positives for each identified turned on appliance combination. As thresholds are not explicitly given for all devices in [49] for our evaluation the decision threshold was empirically selected to 25 W as also in [48,72]. The results are tabulated in Table 9.

As can be seen in Table 9 the $F_{1}$-scores of [49] and of our DTW implementation are almost identical with only 0.5% difference in average, most probably owed to the different preprocessing and threshold settings (the parameter values of them are not given in [49]). In this experiment MVM also outperforms all other elastic matching algorithms and improves the average disaggregation accuracy by 2.4%, resulting in an total average disaggregation accuracy of 89.19% in terms of the $F_{1}$-score. In agreement with the previous evaluations presented in Table 9 sDTW again offers slight performance improvement, while GAK performs slightly worse compared to the baseline DTW, achieving average disaggregation accuracies of 86.84% and 86.26%, respectively. Furthermore, ACS shows a significant performance decrease when compared to DTW, resulting in an average disaggregation accuracy of 79.11%. The highest performance increase is observed for the REDD-1 dataset, improving the energy disaggregation $F_{1}$-score by 4.2% when using MVM as the elastic matching algorithm.

## 6. Conclusions

In this paper an energy disaggregation architecture using elastic matching was presented. In the experimental evaluation five different elastic matching algorithms, namely the dynamic time warping (DTW), the soft-DTW, the global alignment kernel (GAK), the minimum variance matching (MVM) and the all common subsequences (ACS) were evaluated. The experimental results showed that elastic matching algorithms can successfully be used for energy disaggregation, and more specifically it was observed that the minimum variance matching (MVM) algorithm offers the highest energy disaggregation precision both in terms of energy disaggregation accuracy (87.58%) and in terms of $F_{1}$-score (89.19%).

The architecture was evaluated on several datasets with different characteristics and duration, demonstrating that it performs equally well in cases where not many data are available. Specifically the competitive performance of elastic matching-based approach shows that it can offer complementary information to the machine learning-based and the source separation-based NILM approaches, especially in cases when the available data are not enough to train robust NILM models.

## Figures and Tables

**Table 1:** Overview of considered public available datasets and their properties.

Dataset	Parameters				
	#App	#ParaApp	$T_{s}$	*T*	Appliance Type
REDD-1	18	9	3s	14d	One-state/multi-state/ continuous
REDD-2	9	5	3s	11d	One-state/multi-state
REDD-3	20	9	3s	14d	One-state/multi-state/ non-linear
REDD-4	18	8	3s	14d	One-state/multi-state/ continuous/ non-linear
REDD-5	24	11	3s	3d	One-state/multi-state/ non-linear
REDD-6	15	9	3s	12d	One-state/multi-state/ continuous/ non-linear

**Table 2:** Energy disaggregation performance in terms of estimation accuracy ($E_{ACC}$) for different framelengths using dynamic time warping (DTW) as the classifier.

Dataset	Framelength *L*					
	10	25	50	100	200	500
**REDD-1**	74.41%	**76.73%**	73.96%	62.76%	63.60%	60.37%
**REDD-2**	81.88%	**82.31%**	81.37%	79.42%	75.32%	69.34%
**REDD-3**	71.36%	71.80%	71.43%	**72.83%**	71.81%	72.37%
**REDD-4**	83.28%	84.10%	83.39%	84.56%	**84.78%**	78.65%
**REDD-5**	77.71%	**79.56%**	81.25%	78.22%	64.43%	34.29%
**REDD-6**	83.42%	83.13%	82.97%	**83.69%**	83.20%	82.24%
**AVG**	78.67%	**79.61%**	79.06%	76.91%	73.86%	66.21%

**Table 3:** Energy disaggregation performance in terms of $E_{ACC}$ for different restrictions on the DTW warping path.

Dataset	Restrictions on DTW		
	None	Sakoe	Itakura
**REDD-1**	**76.73%**	74.31%	74.20%
**REDD-2**	**82.31%**	79.53%	81.38%
**REDD-3**	**71.80%**	69.88%	71.59%
**REDD-4**	**84.10%**	77.28%	77.97%
**REDD-5**	**79.56%**	74.01%	76.82%
**REDD-6**	**83.13%**	61.66%	60.60%
**AVG**	**79.61%**	72.78%	73.76%

**Table 4:** Energy disaggregation performance in terms of $E_{ACC}$ for different distance metrics’ using DTW.

Dataset	Distance Metric			
	Euclidean	Manhattan	Square	Kullback–Leibler
**REDD-1**	**76.73%**	76.73%	76.68%	76.51%
**REDD-2**	**82.31%**	82.31%	82.19%	81.95%
**REDD-3**	**71.80%**	71.80%	71.57%	71.39%
**REDD-4**	**84.10%**	84.10%	83.40%	83.49%
**REDD-5**	79.56%	79.56%	**80.51%**	80.14%
**REDD-6**	**83.13%**	83.13%	82.28%	82.54%
**AVG**	**79.61%**	79.61%	79.44%	79.34%

**Table 5:** Energy disaggregation performance in terms of $E_{ACC}$ for the free parameters of global alignment kernel (GAK), soft dynamic time warping (sDTW) and minimum variance matching (MVM).

GAK						
γ	**1**	**2**	**5**	**10**	**100**	**500**
	59.44%	64.48%	70.89%	**70.94%**	69.85%	65.74%
**sDTW**						
γ	**1**	**2**	**5**	**10**	**100**	**500**
	72.87%	72.93%	**73.11%**	73.06%	72.06%	69.27%
**MVM**						
**v**	**5**	**10**	**15**	**20**	**25**	**30**
	71.56%	**71.56%**	71.56%	71.56%	71.56%	71.56%

**Table 6:** Energy disaggregation performance in terms of $E_{ACC}$ for different datasets of the reference energy disaggregation dataset (REDD) database using different elastic matching algorithms (average results are provided with and without considering REDD-5).

Dataset	Elastic Matching Algorithm				
	DTW	sDTW	MVM	GAK	ACS
**REDD-1**	73.01%	74.24%	**75.12%**	74.33%	62.63%
**REDD-2**	81.58%	84.65%	**87.58%**	76.45%	71.79%
**REDD-3**	71.67%	72.03%	**73.55%**	72.70%	63.96%
**REDD-4**	80.59%	81.84%	**83.00%**	81.81%	79.17%
**REDD-5**	80.02%	80.19%	**82.13%**	75.75%	63.72%
**REDD-6**	82.24%	80.72%	**84.18%**	82.00%	75.14%
${\mathrm{AVG}}_{1-6}$	78.19%	78.95%	**80.93%**	77.17%	69.40%
${\mathrm{AVG}}_{1,2,3,4,6}$	77.82%	78.70%	**80.69%**	77.46%	70.54%

**Table 7:** Energy disaggregation performance on device level in terms of $E_{ACC}^{m}$ for the REDD-2 dataset using different elastic matching algorithms.

Appliance	Energy Distribution	All Loads					Deferrable Loads				
		DTW	sDTW	MVM	GAK	ACS	DTW	sDTW	MVM	GAK	ACS
**kitchen-outlets**	2.68%	48.84%	49.34%	**59.96%**	54.99%	54.51%	-	-	-	-	-
**lighting**	11.55%	66.23%	69.72%	**74.58%**	25.95%	52.13%	72.12%	81.33%	**82.59%**	74.29%	80.26%
**stove**	0.63%	70.60%	**75.51%**	36.39%	21.37%	38.45%	-	-	-	-	-
**microwave**	6.63%	85.09%	85.32%	**85.80%**	83.33%	59.18%	89.11%	89.32%	89.59%	**90.16%**	71.54%
**washer-dryer**	0.93%	89.03%	89.77%	88.59%	88.99%	81.73%	-	-	-	-	-
**kitchen-outlets**	4.48%	**74.81%**	69.90%	72.94%	52.31%	37.60%	-	-	-	-	-
**refrigerator**	34.48%	82.71%	82.70%	**84.89%**	79.18%	81.18%	93.24%	94.49%	**95.21%**	93.85%	93.17%
**dishwasher**	3.91%	81.94%	**82.61%**	82.52%	77.27%	47.07%	87.25%	86.77%	**89.01%**	88.21%	80.38%
**disposal**	0.03%	**82.51%**	81.22%	81.06%	76.31%	33.10%	-	-	-	-	-
**ghost**	34.98%	85.25%	88.94%	**90.96%**	85.20%	78.41%	-	-	-	-	-
**AVG**	100.00%	81.58%	84.65%	**87.58%**	76.45%	71.79%	88.95%	90.85%	**91.86%**	89.85%	86.24%

**Table 8:** Comparison of $E_{ACC}$ (%) values for recently proposed NILM methodologies (methods marked with an asterisk are not directly comparable because of a dataset transferability setup used in [36] and the reduced number of appliances in [65]).

NILM Method	Publication	Year	Dataset	$E_{\mathrm{ACC}}$	MVM
Greedy Deep SC	[66]	2017	REDD-1/2/3/4/6	62.6%	80.7%
Exact Deep SC	[66]	2017	REDD-1/2/3/4/6	66.1%	
General SC	[67]	2010	REDD-1/2/3/4/6	56.4%	
Discriminating SC	[67]	2010	REDD-1/2/3/4/6	59.3%	
Powerlets-PED	[68]	2015	REDD-1/2/3/4/6	**72.0%**	
Temporal ML	[69]	2011	REDD-1/2/3/4/6	53.3%	
Gibbs Sampling	[70]	2013	REDD-5	55.0%	82.1%
Unsupervised GSP *	[65]	2018	REDD-5	65.0%	
Supervised GSP *	[65]	2018	REDD-5	**79.0**%	
SIQCP	[71]	2016	REDD-2 (deferrable loads)	86.4%	91.9%
Sparse HMM	[56]	2015	REDD-2 (deferrable loads)	**94.8%**	
F-HDP-HSMM	[63]	2013	REDD-2 (deferrable loads)	84.8%	
F-HDP-HMM	[63]	2013	REDD-2 (deferrable loads)	70.7%	
EM-FHMM	[63]	2013	REDD-2 (deferrable loads)	50.8%	
CNN-RNN	[38]	2019	REDD-2 (1 appliance: fridge)	**87.9%**	95.2%
GSP	[65]	2018	REDD-2 (1 appliance: fridge)	85.0%	
CNN *	[36]	2019	REDD-2 (1 appliance: fridge)	83.5%	

**Table 9:** Comparison of DTW proposed in [49] with five different elastic matching algorithms using $F_{1}$-score as defined in Equation (22).

Dataset	Elastic Matching Algorithm					
	DTW [49]	DTW	sDTW	MVM	GAK	ACS
**REDD-1**	82.28%	82.74%	84.95%	**86.85%**	83.68%	74.39%
**REDD-2**	87.04%	88.40%	89.56%	**90.19%**	86.44%	84.38%
**REDD-6**	89.17%	88.82%	86.02%	**90.53%**	88.65%	78.57%
**AVG**	86.16%	86.66%	86.84%	**89.19%**	86.26%	79.11%
